# Ventral tegmental area integrity measured with high-resolution 7-Tesla MRI relates to motivation across depression and anxiety diagnoses

**DOI:** 10.1016/j.neuroimage.2022.119704

**Published:** 2022-10-27

**Authors:** Laurel S. Morris, Marishka Mehta, Christopher Ahn, Morgan Corniquel, Gaurav Verma, Bradley Delman, Patrick R. Hof, Yael Jacob, Priti Balchandani, James W. Murrough

**Affiliations:** a Department of Psychiatry, Icahn School of Medicine at Mount Sinai, New York, USA; b BioMedical Engineering and Imaging Institute, Icahn School of Medicine at Mount Sinai, New York, USA; c Department of Radiology, Icahn School of Medicine at Mount Sinai, New York, USA; d Nash Family Department of Neuroscience and Friedman Brain Institute, Icahn School of Medicine at Mount Sinai, New York, USA

## Abstract

The ventral tegmental area (VTA) is one of the major sources of dopamine in the brain and has been associated with reward prediction, error-based reward learning, volitional drive and anhedonia. However, precise anatomical investigations of the VTA have been prevented by the use of standard-resolution MRI, reliance on subjective manual tracings, and lack of quantitative measures of dopamine-related signal. Here, we combine ultra-high field 400 μm^3^ quantitative MRI with dopamine-related signal mapping, and a mixture of machine learning and supervised computational techniques to delineate the VTA in a transdiagnostic sample of subjects with and without depression and anxiety disorders. Subjects also underwent cognitive testing to measure intrinsic and extrinsic motivational tone. Fifty-one subjects were scanned in total, including healthy control (HC) and mood/anxiety (MA) disorder subjects. MA subjects had significantly larger VTA volumes compared to HC but significantly lower signal intensity within VTA compared to HC, indicating reduced structural integrity of the dopaminergic VTA. Interestingly, while VTA integrity did not significantly correlate with self-reported depression or anxiety symptoms, it was correlated with an objective cognitive measure of extrinsic motivation, whereby lower VTA integrity was associated with lower motivation. This is the first study to demonstrate a computational pipeline for detecting and delineating the VTA in human subjects with 400 *μ*m^3^ resolution. We highlight the use of objective transdiagnostic measures of cognitive function that link neural integrity to behavior across clinical and non-clinical groups.

## 1. Introduction

Dopaminergic projections from the ventral tegmental area (VTA) to the nucleus accumbens (NAc) and medial prefrontal cortex (mPFC), respectively form the mesolimbic and mesocortical pathways in the brain and are important for a range of appetitive, reward-driven behaviors (Hughes et al., 2020; Walsh and Han, 2014). In rodents, activation of the mesolimbic pathway initiates exploratory foraging behavior (DeYoung, 2013; Panksepp and Wilson, 2016), exertion of effort (Salamone et al., 2016) and sustained task engagement (Diederen and Fletcher, 2021). In human subjects with depression, deep brain stimulation of the mesolimbic pathway causes a sudden increase in intrinsic motivation, in terms of increased subjective energy for a previously enjoyable activity (Fenoy and Schiess, 2018; Schlaepfer et al., 2011). Activity of VTA dopamine neurons also has a well-established role in mediating reward prediction error-based learning (Fiorillo et al., 2003; Hooks and Kalivas, 1994; Schultz, 1998), relevant for guiding extrinsic motivation. Therefore, the VTA appears to be a critical region for both intrinsic and extrinsic reward-driven motivation. Given this, structural or functional alterations within the VTA might in part underlie difficulties in intrinsic and extrinsic motivation and reward sensitivity observed in patients, whereby depression and anxiety symptoms relate to disturbances in intrinsic and extrinsic motivation (Barch et al., 2008; Bos et al., 2021; Bowie et al., 2013; Treadway et al., 2009; Yu et al., 2016).

In human studies, measures of the VTA have thus far been limited by relatively low resolution functional and structural MRI procedures. The VTA region in humans comprises multiple specific nuclei, including the parabrachial/parapeduncular nuclei, the linear nuclei and others, which are often included as a general ventral tegmental “area” (Trutti et al., 2019). However, the medial and lateral VTA, which house dopaminergic cell projections to the NAc and mPFC, is a specific bilateral pair of nuclei, with variability in reported size (Eapen and Gore, 2009; Pauli et al., 2018; Trutti et al., 2019). Functional studies remain non-specific to VTA proper, with resolutions around 2 mm with 7 Tesla (7T) MRI (Morris et al., 2019). While recent high-resolution 7T structural MRI studies of the VTA come close to the resolution necessary to delineate the VTA (0.7 mm isotropic) (Trutti et al., 2019), they still include neighboring nuclei within the definition of the VTA and rely on manual drawings which are prone to human error.

To capture the dopamine-rich VTA proper, it is important not only to use high-resolution imaging, but also to harness the capability of 7T MRI for detecting dopaminergic neuron-specific signals, for example by measuring the interaction between large macromolecules and free water exchange via magnetization transfer (MT) MRI (see Priovoulos et al. 2018, 2020) that is likely to indirectly indicate dopamine synthesis and storage capacity of the measured neurons, or dopaminergic cell integrity. MT-MRI captures signal related to neuromelanin, a by-product of catecholamine (dopamine, norepinephrine) synthesis produced by tyrosine hydroxylase. Neuromelanin is concentrated in catecholamine neuron cell bodies in rats (Dahlström, 1964) and human tissue (Nobin and Bjorklund, 1973; van Domburg and ten Donkelaar, 1991) and is linked to high concentrations of cytosolic dopamine in the substantia nigra (Zucca et al., 2014) and VTA (Yamashita et al., 2016). Neuromelanin production by dopamine synthesis can have a protective effect on the cells or, if overexpressed, can indicate cell death, as observed in individuals with Parkinson’s disease (Zucca et al., 2014). Thus, MT-MRI signal intensity can be indicative of dopaminergic cell integrity and previous work has linked this measure of integrity to reward-related learning and motivational flexibility (Chowdhury et al., 2013; Steiger and Bunzeck, 2017). Combined with MT-MRI, it is also important to take advantage of the improved signal separation afforded by high-field MRI as input data for sophisticated machine learning algorithms for fast, robust and data-driven region delineation that does not rely on human perception or manual drawings.

In this study we utilize quantitative high-resolution 400 μm^3^ 7T MT-MRI and machine learning techniques to detect putative dopaminergic neurons in a data-driven manner, to capture VTA in human subjects with and without depression and anxiety disorders. We also collect objective measures of intrinsic and extrinsic motivation (bias) and reward sensitivity (sigma) heuristics (Morris et al., 2020) outside of the scanner in a dimensional manner to examine transdiagnostic relationships between VTA dopaminergic neuronal integrity and motivational tone in humans. We hypothesize that higher VTA dopaminergic neuron integrity is related to higher motivational tone within both intrinsic and extrinsic domains.

## 2. Methods

### 2.1. Participants

Participants aged 18–55 were recruited via the Depression and Anxiety Center for Discovery and Treatment, Icahn School of Medicine at Mount Sinai (ISMMS). Subjects were recruited from web-based advertising, flyers or clinician referrals. Participants with mood and anxiety (MA) disorders were included if they met criteria for major depressive disorder, generalized anxiety disorder, or both, as determined by the Structured Clinical Interview for DSM-5, performed by a trained clinical rater. MA participants were allowed comorbidities of other anxiety, depressive or stress-related disorders, as is highly common in MA subjects (Aina and Susman, 2006; Lamers et al., 2011), but were not included if they had other psychiatric disorders such as obsessive-compulsive disorder, substance or alcohol use disorder, schizophrenia. Healthy control subjects were free of any current or past psychiatric disorder. All subjects were free from a history of current or past neurological disorder, significant head injury, or medications with known antidepressant or anxiolytic effects at the time of the scan. Individuals who met all inclusion and exclusion criteria were enrolled in the study and completed an MRI scan, self-reported measures of symptoms and a cognitive test of intrinsic and extrinsic motivation on the same day. Self-reported symptom measures were performed via a web-based questionnaire system (Redcap) and included demographic information as well as key questionnaires assessing anhedonia: the Temporal Experience of Please Scale (TEPS); and anxiety symptoms: State-Trait Cognitive and Somatic Anxiety (STICSA). General depression was also assessed with the Quick Inventory for Depression Scale (QIDS). The study was approved by the IS-MMS Institutional Review Board, all subjects provided written informed consent and were compensated for their time.

Fifty-one subjects were scanned in total. Of those, 8 were excluded due to technical error, motion or incomplete scanning. Motion was determined via visual inspection of images, judgement of image blurriness and/or visible subject motion during scanning that was not rectified by repeat scan. This left 43 for analysis, comprising 22 HC (age = 30.9 ± 8.6; 7 male) and 21 MA participants (age = 30.5 ± 8.6; 11 male). Of the 21 MA subjects, 11 had primary MDD with 6 of those having a comorbid anxiety disorder, and 10 had primary GAD with 7 having a comorbid depressive disorder, as determined by the SCID. See Table 1 for full participant details.

### 2.2. Image acquisition, processing and machine learning analysis

All participants underwent 7T MRI scanning (Magnetom, Siemens, Erlangen, Germany) with a 32-channel transmit coil at the BioMedical Engineering and Imaging Institute (BMEII), ISMMS, New York. A T1-weighted structural dual-inversion magnetization prepared gradient echo (MP2RAGE) scan was acquired with whole brain coverage for segmentation and alignment purposes, using the following parameters: TR = 4500 ms, TE = 3.37 ms, TI1 = 1000 ms and 3200 ms, flip = 4 and 5°, iPAT acceleration factor = 3, bandwidth = 130 Hz/pixel, 700 μm^3^ resolution (Marques et al., 2010). Next, an MT-MRI scan was collected (see Priovoulos et al. 2018) with an oblique slab spanning the ventral tegmentum of the midbrain and pons using a 3D segmented GRE turbo-FLASH (TR = 1180 ms, TE = 3.61 ms, flip = 8°, frames = 56, field of view = 448 × 448, slice thickness = 0.5 mm, pixel bandwidth = 140, pixel spacing = 0.43 × 0.43), with 20 MT pulses (190 V transmit) and 7 min scan duration, providing 400 *μ*m isotropic resolution, high signal-noise-ratio for the midbrain and higher efficiency in time and SAR compared to other methods (e.g. T1-TSE). Next, an identical MT-TFL MRI scan was acquired with identical slab placement and resolution, but without MT pulses (i.e., non-MT) with 4 min scan time for computation of signal enhancement related to dopaminergic cell integrity.

MT images were coregistered to the Freesurfer-segmented T1-weighted anatomical image with a boundary-based technique. Similar to Morris et al. (2020), the MT image was divided by the non-MT image which generated an MT enhancement image, indicating voxels whose signal intensity is mediated by presence of large macromolecules such as neuromelanin as a by-product of dopamine synthesis.

The enhancement image was subjected to Gaussian Mixture Modeling (GMM) with Scikit-learn in Python (Pedregosa et al., 2011) to learn, sample and estimate Gaussian Mixture Models from the data, assuming that data are generated from a mixture of finite Gaussian distributions with unknown parameters, incorporating information about both the variance structure and the latent center points. Similar to previous work (Morris et al., 2020), the number of components was set to three in order to classify voxels into neuromelanin-related, white matter and cerebrospinal fluid components. The expectation–maximization algorithm was implemented for model parameter estimation and to compute and update the weights of the latent model variables to maximize likelihood based on all data points.

As expected and similar to our previous work (Morris et al., 2020), the neuromelanin-related classification cluster revealed dopamine-rich structures including substantia nigra (SN) and ventral tegmentum in a data-driven manner without manual tracing. VTA segmentations were visually inspected in relation to Nissl-based anatomy as defined by the Allen Institute for Brain Science Human Brain Atlas (Ding et al., 2016). The SN was delineated and utilized as a control region for validation purposes given its rich dopaminergic neuron composition, large size, close proximity, and distinctly visible signal intensity boundaries important for visual validation. Next, a 4-cluster GMM model was computed with the same model parameters to separate the neuromelanin-related classification cluster into distinct subtle signal intensity contributions expected from VTA (ventral, medial) versus SN proper. The putative VTA was anatomically restricted along the dorsal-ventral boundary as falling within the ‘midbrain’ Freesurfer segmentation and the medial-lateral boundary as parallel to the 4th ventricle. The resultant data-driven delineation of VTA was visually inspected by expert neuroanatomists (PRH, BD) and compared to precise cytoarchitectural whole-brain reconstructions (Ding et al., 2016). VTA delineations were thus completed for each subject, visually inspected, converted to volume computations and the neuromelanin-related signal intensity was calculated within each individual VTA volume. A group mean VTA volume was computed for the HC group. VTA volume and signal intensity measures for each subject were finally normalized against whole brain volume measures for each individual.

### 2.3. Internal-external motivation task

The Internal-external Motivation Task (IMT) (Morris, et al., 2020) is a cognitive effort-based decision-making task for physical effort, programmed with PsychoPy software. There are two conditions testing internally-generated motivation (related to intrinsic motivation) and externally-generated motivation (related to extrinsic motivation). In the external condition, subjects must accept or reject offers of various levels of physical effort (between 3 and 70 button presses) and monetary reward ($0.25 to $2.00). In the internal condition, subjects must self-generate the level of effort they would be willing to work for the same levels of rewards. During training, subjects were instructed that the more effort they agree to, the more likely they are to be awarded the money. In actuality, all subjects received the maximum bonus of $2.00. During this task, a random thirty percent of trials lead to the work, all trials are interleaved, response times are self-paced and subjects are instructed on the task in a self-paced manner before starting. Similar to previous studies (Klein-Flügge et al., 2015; Morris, et al., 2020), a psychometric sigmoid function was fit to the effort-by-reward discount curves for each condition for each subject using the variational Bayes approach to model inversion implemented in the VBA toolbox (available at mbb-team.github.io/VBA-toolbox), under a mixed-effects framework, using MATLAB R2019a. See supplementary methods for further details and supplementary Fig. 2 for model fits. This approach provides a bias parameter which represents the left-right translation of the function, henceforth referred to as “motivational tone” whereas sigma governs reward sensitivity or gradient (see Morris, et al. 2020) for additional details). These parameter estimates were derived for each condition for each subject and normalized via z-score normalization.

### 2.4. Statistics

Since VTA signal intensity was expected to be the main proxy measure of dopaminergic neuron integrity, VTA signal intensity was primarily examined for group differences and in relation to the dimensional objective motivational measures and self-reported symptom measures. Normalized VTA signal intensity values were entered into independent samples t-test to initially compare between groups and linear regression analysis for the full cohort, to examine relationships with the objective normalized cognitive measures of motivational tone (bias) and reward sensitivity (sigma), controlling for sex. Separately, VTA signal intensity was correlated against self-reported measures of anticipatory and consummatory anhedonia (Temporal Experience of Please Scale, TEPS), and normalized anxiety symptoms (State-Trait Cognitive and Somatic Anxiety, STICSA total score) across the whole cohort primarily, with follow-up analyses within the MA group alone.

## 3. Results

### 3.1. VTA segmentation

**Fig. 1.**
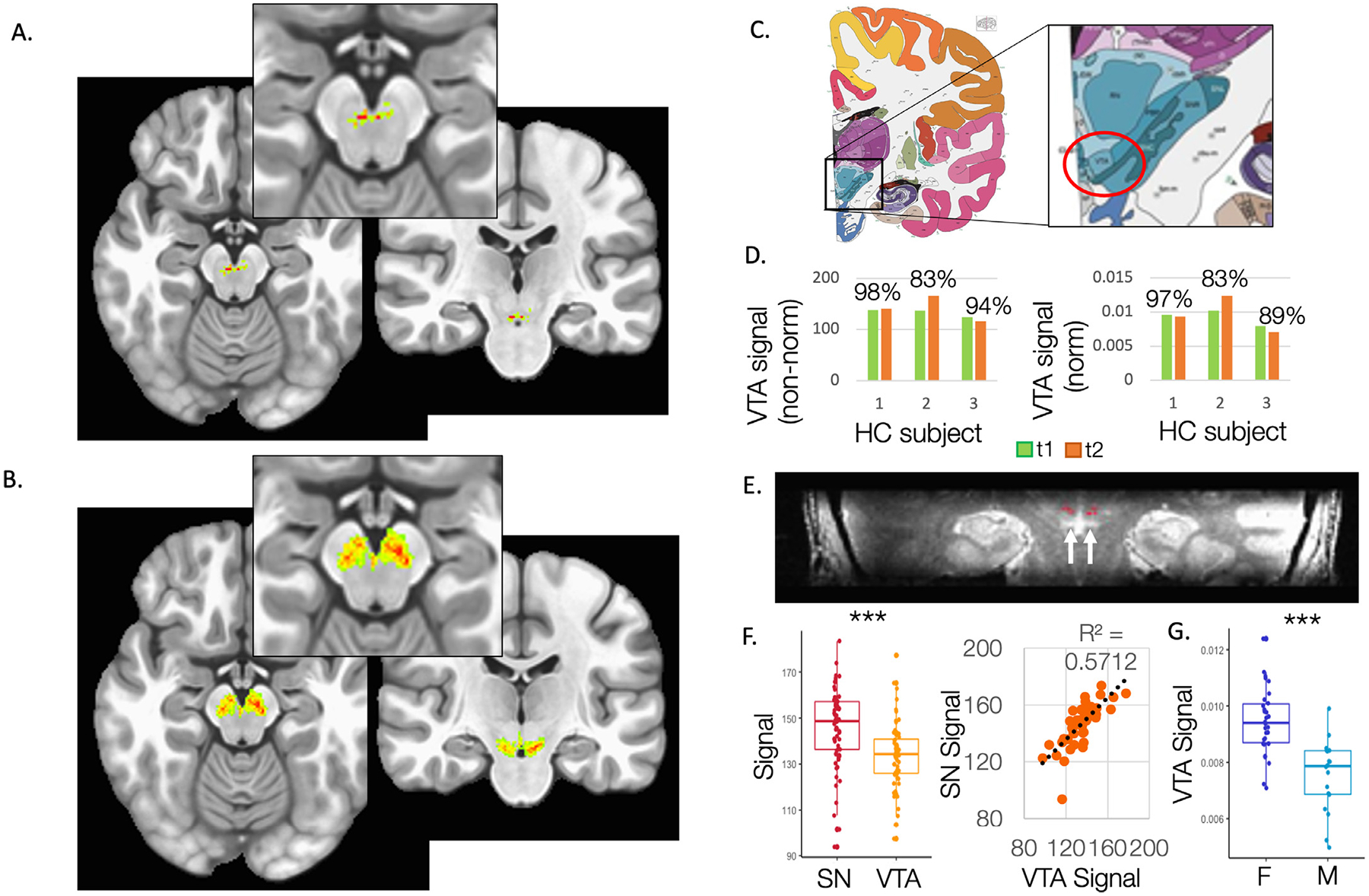
Machine-learning data-driven segmentations of ventral tegmental area and substantia nigra. A. Composite group image of VTA segmentations produced by a mixture of supervised and unsupervised models based on high-resolution 7-Tesla 400 um^3^ magnetization transfer contrast MRI. B. Composite group image of SN segmentations following the same procedure. C. Whole brain structural delineations from the Allen Institute for Brain Science Human Brain Atlas (Ding et al., 2016), with VTA highlighted. D. Intra-subject test-retest reliability for 3 healthy control subjects scanned twice, at time 1 (t1) and time 2 (t2), with percentage overlap indicated. E. A random healthy control single subject VTA delineation. F. Signal intensity for the two neighboring regions of VTA and SN and their correlation. G. VTA signal intensity plotted for females (F) and males (M) across the cohort. *** *p* < 0.001

Across the HC subjects, VTA segmentations showed overlap between subjects and followed the Nissl-based anatomical delineations of VTA as defined by the Allen Institute for Brain Science Human Brain Atlas (Fig. 1A–C; (Ding et al., 2016), see also Supplementary Fig. 1), with up to 97% intra-subject test-retest reliability (Intraclass Correlation Coefficient = 0.705 for 3 HC subjects scanned twice, Fig. 1D), and resolvable single-subject volumes (Fig. 1E). Despite high correlation in signal intensity between the two neighboring regions of VTA and SN (Fig. 1F), VTA signal intensity was lower than that of the control region, SN (*t* = −3.738, *p* = 0.0003, Fig. 1F), as expected given the larger proportion of dopamine neurons in SN compared to VTA (Nair-Roberts et al., 2008; Reyes et al., 2012). VTA signal intensity was also higher in females compared to males across the cohort (*t* = −5.172, *p* = 1.07 × 10^−5^, Fig. 1G), in line with evidence of higher dopamine neuron expression in VTA for female compared to male rats (Bangasser and Cuarenta, 2021; Kokane and Perrotti, 2020). There was no correlation with age (*R* = 0.106, *p* = 0.474). Finally, raw values of VTA volumes and signal intensity were not correlated with each other or whole-brain volume measures (p’s > 0.05) suggesting that the volume was not computed simply due to variability in signal intensity.

### 3.2. Group differences

**Fig. 2.**
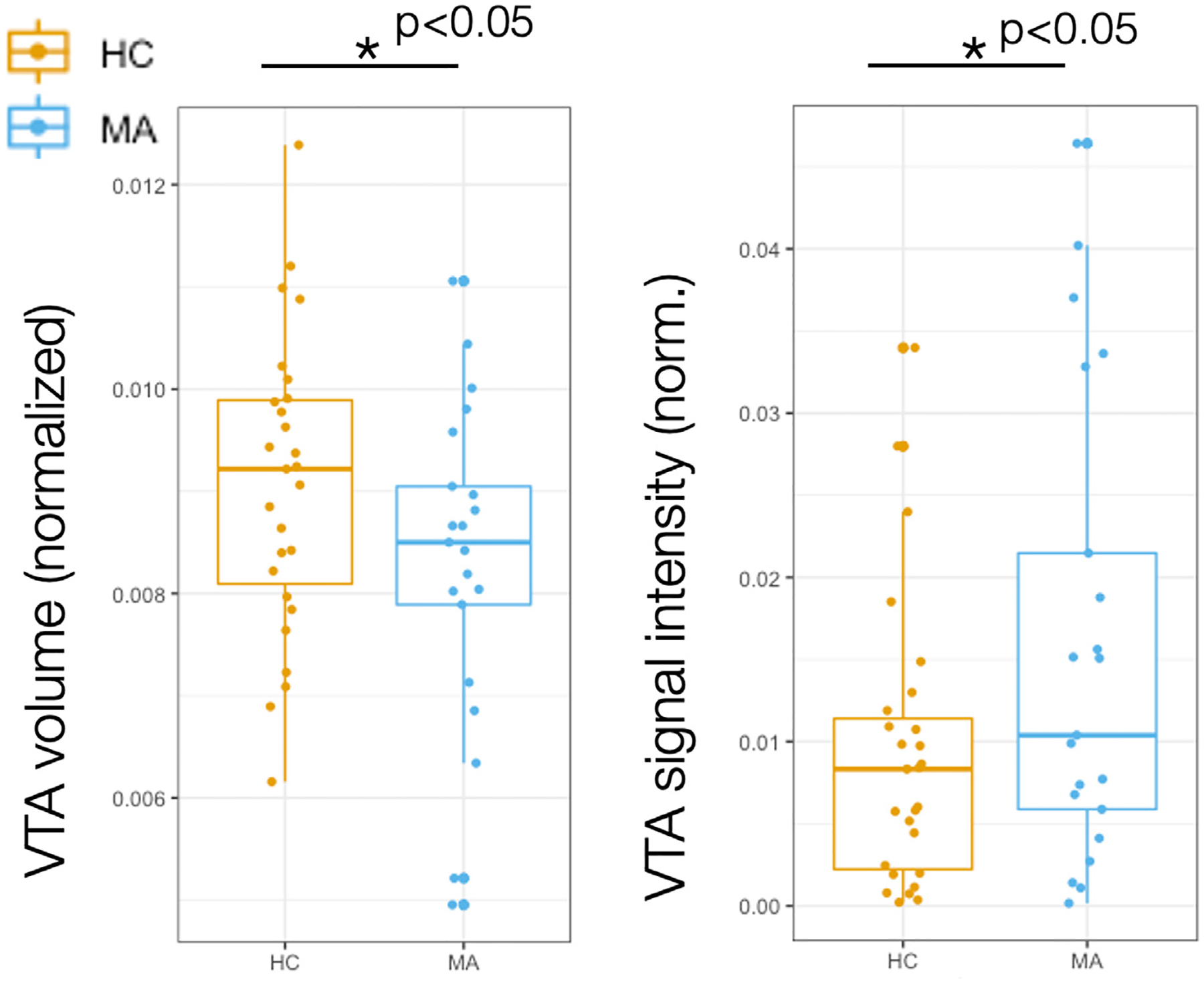
Subjects with depression and anxiety show higher ventral tegmental area volume but reduced neuronal integrity compared to controls. Group differences of normalized (norm) ventral tegmental area (VTA) structural metrics between healthy control (HC) subjects and subjects with depression and mood (MA) disorders. There was larger normalized VTA volume for MA compared to HC (*t* = 2.037, *p* = 0.023), but lower VTA signal intensity for MA compared to HC (*t* = 1.71, *p* = 0.047).

DA subjects had larger normalized VTA volumes compared to HC (*t* = 2.037, *p* = 0.023), and lower signal intensity within VTA compared to HC (*t* = 1.71, *p* = 0.047) (Fig. 2), suggesting reduced structural integrity of the VTA in participants with MA disorders. Similar findings were apparent when using non-normalized (‘raw’) values of VTA volume (i.e., cluster size, *t* = 2.039, *p* = 0.049) and signal intensity (*t* = 1.631, *p* = 0.11). While VTA signal intensity was highly correlated with the control region SN signal intensity (*R* = 0.779, *p* = 7.9 × 10^−10^), there were no differences in SN signal intensity between groups (*t* = 0.555, *p* = 0.583), indicating specificity in group differences for the VTA.

### 3.3. Correlations with objective and subjective symptom measures

**Fig. 3.**
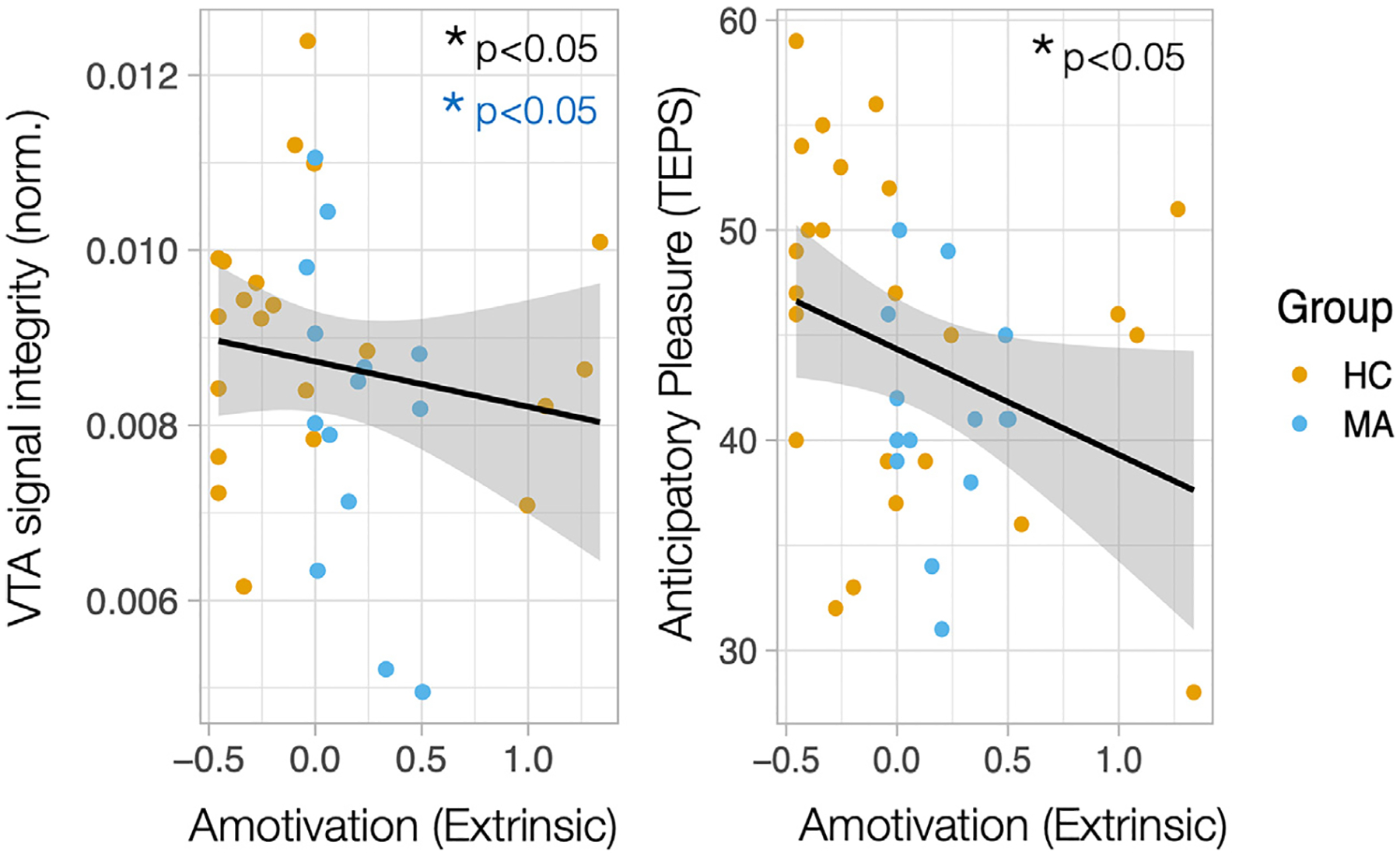
Ventral tegmental area integrity is related to motivational tone. Correlations between ventral tegmental area (VTA) signal intensity and an objective cognitive measure of extrinsic motivation is plotted across the cohort, including heathy control (HC) subjects (orange) and individuals with mood and anxiety (MA) disorders (blue). Extrinsic motivation is plotted against anticipatory anhedonia across the cohort.

VTA signal intensity, the major proxy measure of dopaminergic cell integrity, was primarily examined for relationships with objective and subjective symptom measures dimensionally across groups controlling for sex. VTA signal intensity did not significantly correlate with self-reported anhedonia or anxiety symptoms across the cohort (p’s > 0.05), or within the MA group alone. However, VTA signal intensity was significantly correlated with the objective cognitive measure of extrinsic motivation (bias) dimensionally across the cohort (*R* = 0.407, *p* = 0.041) (Fig. 3), whereby lower VTA integrity was associated with lower extrinsic motivation. Adding *group* to an exploratory general linear model marginally reduced this significance (effect of bias: *t* = −1.93, *p* = 0.063; effect of group: *t* = −0.652, *p* = 0.519; overall adjusted R^2^ = 0.396, *F* = 8.437, *p* = 0.003). The correlation between VTA signal intensity and bias was significant for the MA disorder group alone (*R* = 0.467, *p* = 0.046). Conversely, VTA signal intensity was not related to dimensional measures of reward sensitivity across the cohort (p’s > 0.05).

**Fig. 4.**
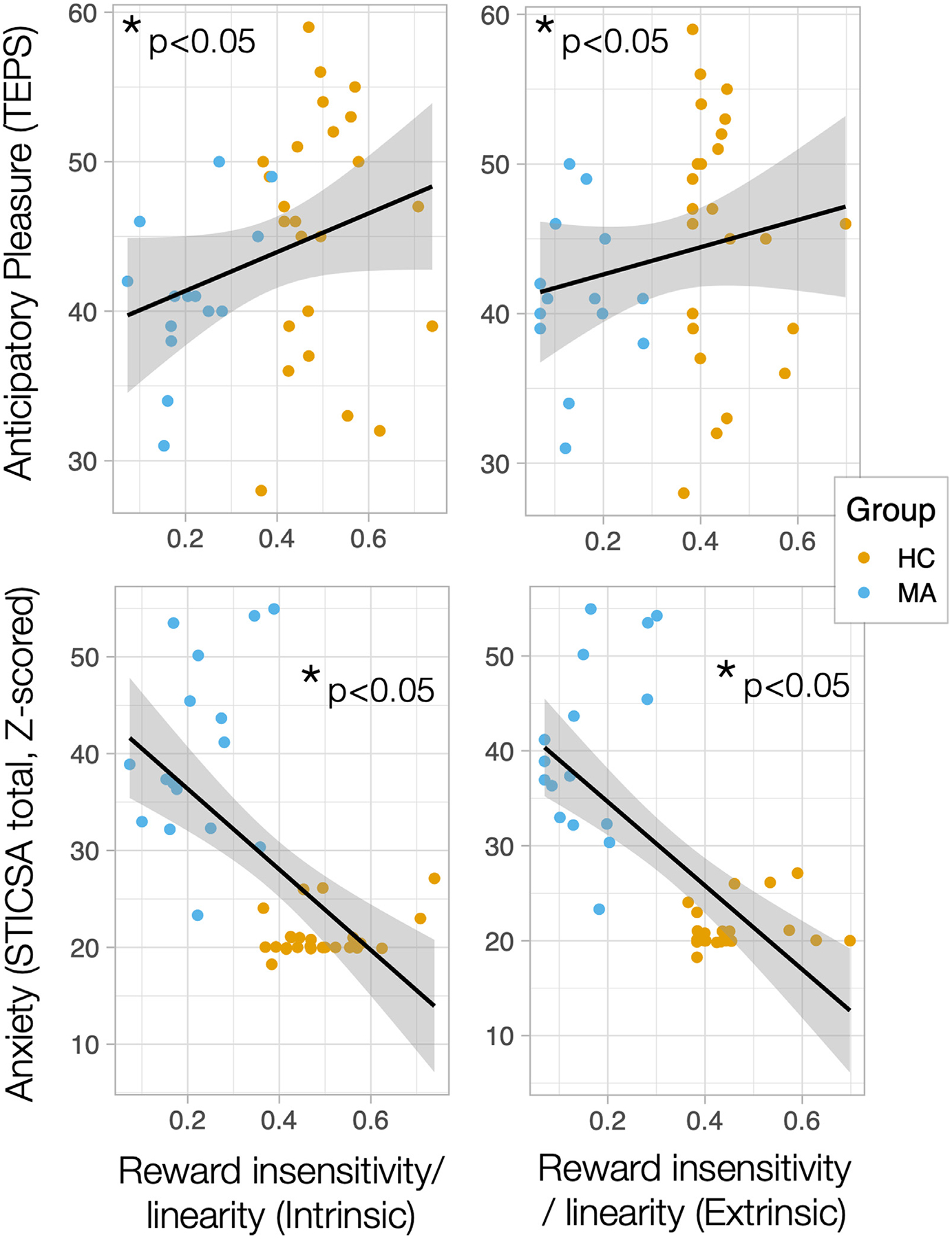
Relationships between reward sensitivity and symptoms. Self-reported symptoms of anticipatory anhedonia and anxiety (Z-scored) are plotted against reward sensitivity for both intrinsic and extrinsic conditions across the cohort, including heathy control (HC) subjects (orange) and individuals with mood and anxiety (MA) disorders (blue).

There were no differences in motivation measures (bias) between groups (see Table 1), although self-reported symptoms of anticipatory anhedonia (Spearman rho = −0.387, *p* = 0.016, Fig. 4) and anxiety (Spearman rho = 0.565, *p* = 0.0001) were associated with extrinsic motivation, whereby worse symptoms were associated with lower motivation. In contrast, there were significant differences in reward sensitivity (sigma) between groups (see Table 1), whereby MA subjects showed more binary decision-making than controls. Self-reported symptoms of anxiety were associated with reward sensitivity for both intrinsic (Spearman rho = −0.661, *p* = 2.6 × 10^−6^) and extrinsic (Spearman rho = −0.674, *p* = 1.4 × 10^−6^) conditions across the cohort, whereby more binary decision-making was associated with worse symptoms (Fig. 4). Anticipatory anhedonia was also associated with intrinsic reward sensitivity (sigma, Spearman rho = 0.321, *p* = 0.049), whereby worse anhedonia was associated with more binary decision-making. However, these correlations did not hold for the MA group alone (p’s > 0.1), suggesting that they might be driven by group differences in both symptom measures and motivation measures. There were no sex differences in motivational tone (bias) or reward sensitivity (sigma) parameters (p’s > 0.05). See Supplementary Table 1 for all correlations.

## 4. Discussion

This work sought to develop and apply a machine-learning based approach to the delineation of the VTA based on ultra-high field 7T MRI images that indicate dopamine synthesis capacity and dopaminergic cell integrity in humans. In parallel, specific VTA structural features were linked with objective cognitive measures of intrinsic and extrinsic motivation (bias) and reward sensitivity, across patients with and without depression and anxiety disorders. Overall, VTA cell integrity was reduced in the clinical population compared to controls, and lower VTA integrity was associated with lower extrinsic motivation dimensionally. This is the first known use of high-field quantitative magnetization transfer contrast imaging of the VTA in subjects with mood and anxiety disorders.

Machine learning and deep learning tools are increasingly being used in medical imaging to more accurately and rapidly detect lesions, abnormal tissue or other artefacts based on training data (Hale et al., 2018; Hasan et al., 2019; Mehrotra et al., 2020). This not only removes burden from radiologists, but also provides higher precision compared to human efforts (Hasan et al., 2019). However, these applications also come with challenges in that machine learning classifications can be biased based on training data that are used for model development. In this study, no training data was used and the machine learning - based Gaussian mixture model was able to distinguish tissue properties directly, based on neuronal cell composition putatively related to dopamine synthesis capacity. Thus, utilizing physical information from images that is biologically-informed will allow for less biased classifications. These computerized methods might also provide additional benefit when based on 7T MRI data that has greater information but more subtle variations that cannot be detected with the human eye due to the vastly improved spatial resolution. The benefit of this combination of high-resolution MRI data with machine-learning based signal detection was confirmed in the current study since the findings overlapped well with histological delineations of the VTA (Ding et al., 2016), produced expected patterns of signal intensity relative to neighboring structures such as SN, provided individualized segmentations for all subjects including psychiatric groups, had good within-subject consistency and was performed within a rapid computational framework. Together, this indicates the widespread applicability of this combination of tools.

This study implicated relationships between VTA dopaminergic neuron integrity and an objective measure of extrinsic motivation, rather than intrinsic motivation across all subjects. The phasic and tonic firing of VTA dopaminergic neurons is implicated in representing reward anticipation, across multiple time scales by a growing literature (Bromberg-Martin et al., 2010; Kim et al., 2020). The role of the VTA in reward-prediction error (Fiorillo et al., 2003; Schultz, 1998), temporal difference learning (Starkweather and Uchida, 2021) and overall salience perception of external stimuli (Kutlu et al., 2021), clearly indicates a role for VTA dopaminergic activation in extrinsic reward-related learning. Despite evidence that VTA dopamine firing does not ramp up with state value during reward approach (Mohebi et al., 2019) more recent reports demonstrate a pattern of VTA dopaminergic neuron firing that does increase during reward approach but is consistent with reward-prediction error, rather than reward value per se (Kim et al., 2020), implicating the VTA in reward-related prediction rather than value estimation. A recent report (Hughes et al., 2020) demonstrated increased VTA dopaminergic firing that scales with force exerted during reward approach behavior, but only in the context of reward, further implicating VTA dopaminergic firing in extrinsic reward-driven motivated behavior. However, these studies indicate a link with phasic VTA dopamine neuron activity, which is difficult to capture in humans with structural MRI. Slower, tonic release of dopamine modulates dopaminergic cell tone, and neuromelanin might act as an indicator of dopamine concentrations that are more related to tonic cell activity (Rusheen et al., 2020). As such, the current methods might indicate tonic cell tone, but they do not afford insight into phasic activity that encodes important reward-related motivational aspects of behavior.

The question of intrinsic reward-related motivation is more elusive and understudied (Morris et al., 2022). A recent neurocomputational study modeling intrinsic reward as “achievement” or the successful completion of a difficult challenge, found neural activation in the ventromedial prefrontal cortex (vmPFC) related to intrinsic value (Chew et al., 2021), with no evidence of signaling in the VTA. However, this study was performed with 3T imaging and may not have had the optimal resolution for measuring VTA activity. Nonetheless, there are extensive mesocortical dopaminergic projections from the VTA to the vmPFC and surrounding medial frontal regions (Haber and Knutson, 2010; Walsh and Han, 2014) that might mediate value learning for all rewards, whether intrinsic or extrinsic (Chew et al., 2021; Haber and Knutson, 2010; Murayama et al., 2010, 2015; Tzschentke, 2000). Other neural structures targeted by VTA dopaminergic projections are also critical to reward-related effort exertion, such as the NAc and anterior cingulate cortex (Klein-Flügge et al., 2016; Müller et al., 2021). Further work must be done to measure the role of VTA and the wider mesolimbic/mesocortical circuitry in reward learning for intrinsic versus extrinsic rewards, and how that translates into motivational vigor.

The mesolimbic/mesocortical circuitry is considered to be critical to the pathophysiology of depression and anxiety disorders. In rodent models of depression and anxiety, VTA hyperactivity and hyperconnectivity has been demonstrated and link to anhedonic phenotypes (Cao et al., 2010; Chaudhury et al., 2013; Coque et al., 2011; Morris et al., 2019) while reducing VTA hyperconnectivity normalizes depressive, anhedonic behaviors (Friedman et al., 2016). Animal studies have shown disruption of the phasic firing of the VTA neurons and subsequent activation of NAc in stress-susceptible mice with depressive symptoms (Chaudhury et al., 2013). The hyperactivity of these neurons is also associated with anxiety-like symptoms, with reduced GABAergic regulation from the NAc (Qi et al. 2022). Interestingly, and in line with the current work, female rodents have higher dopamine content in the VTA compared to males (Bangasser and Cuarenta, 2021; Kritzer and Creutz, 2008; McArthur et al., 2007) and stress impacts VTA function differently in females compared to males (Bangasser and Cuarenta, 2021; Rincón-Cortés and Grace, 2017). Because a reduction in neuromelanin in dopaminergic neurons can be indicative of cell death related to excitotoxicity as observed in Parkinson’s disease (Knörle, 2018), the current work might provide a proxy indication of cell damage caused by excessive VTA hyperactivity, which needs be explored and validated in future work. Together, these studies emphasize the importance of the VTA circuitry in mediating goal-directed, motivated behavior, which form core characteristics of anhedonic symptoms of depression.

While some studies do not distinguish between subnuclei of the SN in terms of MT-MRI signal (Zecca et al., 2003), MT-MRI is likely more favorably capturing the SN pars compacta (SNc), which contains higher numbers of dopaminergic neurons and allows neuromelanin accumulation, whereas the SN pars reticulata (SNr) contains higher levels of iron that can be detected more readily via susceptibility-weighted imaging (Langley et al., 2015; Lehéricy et al., 2014). The medial and lateral subregions of the VTA have also been shown to have distinct functions related to reward sensitivity, learning, as well as in their innervation targets (Beier et al., 2015; Ikemoto, 2007; Lammel et al., 2014). Follow-up work using high-resolution MRI of the VTA should explore the utility of voxelwise or clustering analysis of the VTA, similar to recent work parsing distinct subzones of small brainstem structures (Jacobs et al., 2022), to parse VTA subdivisions that might underlie distinct phenotypes.

A number of limitations to this study should be noted. First, only volunteer unmedicated subjects were included to study the VTA and the neural correlates of motivation and anhedonia without the confounds of medication. This biases the sample to a milder presentation of depression and anxiety disorders, who are motivated enough to join a research study, reducing the generalizability of the findings. Further studies in a larger sample of both medicated and unmedicated subjects who might be more severe, or at least incorporate a range of phenotypes, will allow more specific parcellation of potential biomarkers. Second, while the cognitive task of motivation separates out self-generated motivation from externally-generated motivation, rewards for both forms of motivation were monetary gains, which is an extrinsic reinforcer. Therefore, while the internal condition of the task might indicate intrinsic motivation broadly, further studies that do not use monetary rewards would provide a close approximation of intrinsic motivation. Third, this study was largely hypothesis-driven in a relatively small cohort, and larger studies with rigorous corrections for multiple comparisons will be required to validate these findings. Fourth, while 7T MRI provides higher spatial resolution for exploring small brainstem nuclei, not all facilities have a 7T MRI scanner available and not all individuals are suitable for 7T MRI scanning. Thus, more feasible lower-field 3T imaging techniques must be developed and optimized for best applicability to a broader population, as well as optimization of other more commonly used modalities such as T1-weighted imaging. Finally, while several studies have used MT-MRI for examining the VTA (van Domburg and ten Donkelaar, 1991; Yamashita et al., 2016), some previous studies have struggled to identify the VTA with this technique (Watanabe et al., 2014), possibly due to the lower neuromelanin concentration in VTA as observed in this study, due to the lower concentration and proportion of dopamine neurons in VTA compared to the substantia nigra. This suggests that these subtle differences in neuromelanin concentrations in the VTA compared to surrounding nuclei and white matter might be better localized by computational algorithms to detect small signal differences that might not be visible via the naked eye. Further, there has been speculation around the source of the MT-MRI signal, seemingly being more related to a higher ratio of free water versus large macromolecules such as neuromelanin, rather than neuromelanin directly (Priovoulos et al., 2020). Certainly, future studies that combine high-resolution *ex vivo* MRI with cytoarchitectural mapping or cell staining of post-mortem human neuronal sections will be critical in determining more direct biological nature of MRI signal properties.

## 5. Conclusion

This study demonstrates a machine learning -based computational pipeline for detecting and delineating the VTA in human subjects with 400 *μ*m^3^ resolution. Subjects with MA disorders showed reduced structural integrity of the VTA, associated with reduced motivation. We highlight the use of objective transdiagnostic measures of neural and cognitive function that link neural integrity to behavioral function across clinical groups.

## Supplementary Material

1

2

3

## Figures and Tables

**Table 1:** Subject characteristics. Demographic and self-report data for healthy control (HC) subjects and subjects with depression and anxiety disorders. Anhedonia was measured via the Temporal Experience of Please Scale (TEPS), anxiety via the State-Trait Cognitive and Somatic Anxiety (STICSA) total score and depression via the Quick Inventory for Depression Scale (QIDS).

	Healthy Control	Depression and Anxiety	p value
N	22	21	
Age (mean ± SD)	31.37 ± 8.92	30.73 ± 8.84	0.816
Females (frequency, %)	15, 68.18%	10, 47.62%	0.172
Hispanic ethnicity (frequency, %)	1, 4.55%	4, 19.04%	0.138
Employed, at least part-time (frequency, %)	16, 72.72%	17, 80.95%	0.523
Some college (frequency, %)	22, 100%	19, 90.48%	0.138
Married (frequency, %)	5, 22.73%	3, 14.29%	0.477
Anxiety	22.91 ± 2.37	38.57 ± 10.06	<0.001
Cognitive	11.32 ± 1.96	21.76 ± 6.65	<0.001
Somatic	11.59 ± 0.91	16.81 ± 4.96	<0.001
Anhedonia (Anticipatory)	46.86 ± 8.22	40.25 ± 6.62	0.007
Anhedonia (Consummatory)	37.86 ± 6.73	36.63 ± 9.25	0.540
Depression	1.47 ± 1.32	11.13 ± 4.67	<0.001
External Bias (Amotivation)	0.034 ± 0.57	0.193 ± 0.57	0.142
Internal Bias (Amotivation)	0.545 ± 0.46	0.483 ± 0.42	0.315
External Sigma (Reward Sensitivity)	0.450 ± 0.09	0.159 ± 0.09	1.21 × 10^−13^
Internal Sigma (Reward Sensitivity)	0.491 ± 0.10	0.222 ± 0.10	3.39 × 10^−11^

## Data Availability

Data will be made available on request.

## Data sharing and availability

Data may be made available via request. Data sharing will require a formal data sharing agreement and approval from ethics committees involved. Scripts and software will be made freely available upon request.

## References

[R1] Aina Y, Susman JL, 2006. Understanding comorbidity with depression and anxiety disorders. J. Am. Osteopath. Assoc 106 5 SUPPL.. 16738013

[R2] Bangasser DA, Cuarenta A, 2021. Sex differences in anxiety and depression: circuits and mechanisms. Nat. Rev. Neurosci 22 (11). doi:10.1038/s41583-021-00513-0. 34545241

[R3] Barch DM, Yodkovik N, Sypher-Locke H, Hanewinkel M, 2008. Intrinsic motivation in schizophrenia: relationships to cognitive function, depression, anxiety, and personality. J. Abnorm. Psychol 117 (4). doi:10.1037/a0013944. 19025225

[R4] Beier KT, Steinberg EE, Deloach KE, Xie S, Miyamichi K, Schwarz L, Gao XJ, Kremer EJ, Malenka RC, Luo L, 2015. Circuit architecture of VTA dopamine neurons revealed by systematic input-output mapping. Cell 162 (3). doi:10.1016/j.cell.2015.07.015. PMC4522312 26232228

[R5] Bos DJ, Barnes ED, Silver BM, Ajodan EL, Clark-Whitney E, Scult MA, Power JD, Jones RM, 2021. An effort-based social feedback paradigm reveals aversion to popularity in socially anxious participants and increased motivation in adolescents. PLoS One 16. doi:10.1371/journal.pone.0249326, 4 April 2021. PMC8078767 33905429

[R6] Bowie CR, Gupta M, Holshausen K, Jokic R, Best M, Milev R, 2013. Cognitive remediation for treatment-resistant depression: effects on cognition and functioning and the role of online homework. J. Nerv. Ment. Dis 201 (8). doi:10.1097/NMD.0b013e31829c5030. 23896849

[R7] Bromberg-Martin ES, Matsumoto M, Hikosaka O, 2010. Dopamine in motivational control: rewarding, aversive, and alerting. Neuron 68 (5). doi:10.1016/j.neuron.2010.11.022. PMC3032992 21144997

[R8] Cao JL, Covington HE, Friedman AK, Wilkinson MB, Walsh JJ, Cooper DC, Nestler EJ, Han MH, 2010. Mesolimbic dopamine neurons in the brain reward circuit mediate susceptibility to social defeat and antidepressant action. J. Neurosci 30 (49). doi:10.1523/JNEUROSCI.3177-10.2010. PMC3061337 21147984

[R9] Chaudhury D, Walsh JJ, Friedman AK, Juarez B, Ku SM, Koo JW, Ferguson D, Tsai HC, Pomeranz L, Christoffel DJ, Nectow AR, Ekstrand M, Domingos A, Mazei-Robison MS, Mouzon E, Lobo MK, Neve RL, Friedman JM, Russo SJ, Han MH, 2013. Rapid regulation of depression-related behaviours by control of midbrain dopamine neurons. Nature 493 (7433). doi:10.1038/nature11713. PMC3554860 23235832

[R10] Chew B, Blain B, Dolan RJ, Rutledge RB, 2021. A neurocomputational model for intrinsic reward. J. Neurosci 41 (43). doi:10.1523/JNEUROSCI.0858-20.2021. PMC8549542 34544831

[R11] Chowdhury R, Guitart-Masip M, Lambert C, Dolan RJ, Düzel E, 2013. Structural integrity of the substantia nigra and subthalamic nucleus predicts flexibility of instrumental learning in older-age individuals. Neurobiol. Aging 34 (10). doi:10.1016/j.neurobiolaging.2013.03.030. PMC3713434 23623600

[R12] Coque L, Mukherjee S, Cao JL, Spencer S, Marvin M, Falcon E, Sidor MM, Birnbaum SG, Graham A, Neve RL, Gordon E, Ozburn AR, Goldberg MS, Han MH, Cooper DC, McClung CA, 2011. Specific role of VTA dopamine neuronal firing rates and morphology in the reversal of anxiety-related, but not depression-related behavior in the clock*δ*19 mouse model of mania. Neuropsychopharmacology 36 (7). doi:10.1038/npp.2011.33. PMC3096816 21430648

[R13] Dahlström AFK, 1964. Evidence for the existence of monoamine-containing neurons in the central nervous system. Acta Physiol. Scand. 14229500

[R14] DeYoung CG, 2013. The neuromodulator of exploration: a unifying theory of the role of dopamine in personality. Front. Hum. Neurosci doi:10.3389/fnhum.2013.00762, NOV. PMC3827581 24294198

[R15] Diederen KMJ, Fletcher PC, 2021. Dopamine, prediction error and beyond. Neuroscientist 27 (1). doi:10.1177/1073858420907591. PMC7804370 32338128

[R16] Ding SL, Royall JJ, Sunkin SM, Ng L, Facer BAC, Lesnar P, Guillozet-Bongaarts A, McMurray B, Szafer A, Dolbeare TA, Stevens A, Tirrell L, Benner T, Caldejon S, Dalley RA, Dee N, Lau C, Nyhus J, Reding M, Lein ES, 2016. Comprehensive cellular-resolution atlas of the adult human brain. J. Comp. Neurol 524 (16). doi:10.1002/cne.24080. PMC5054943 27418273

[R17] Eapen M, Gore JC, 2009. Identifying the functional architecture of the human ventral tegmental area and the substantia nigra using high resolution magnetic resonance imaging. Neurosci. Vanderbilt Rev 1 (May).

[R18] Fenoy AJ, Schiess MC, 2018. Comparison of tractography-assisted to atlas-based targeting for deep brain stimulation in essential tremor. Mov. Disord 33 (12). doi:10.1002/mds.27463. 30187527

[R19] Fiorillo CD, Tobler PN, Schultz W, 2003. Discrete coding of reward dopamine neurons. Science 299 (March). 10.1126/science.1077349 12649484

[R20] Friedman AK, Juarez B, Ku SM, Zhang H, Calizo RC, Walsh JJ, Chaudhury D, Zhang S, Hawkins A, Dietz DM, Murrough JW, Ribadeneira M, Wong EH, Neve RL, Han MH, 2016. KCNQ channel openers reverse depressive symptoms via an active resilience mechanism. Nat. Commun 7. doi:10.1038/ncomms11671. PMC4890180 27216573

[R21] Haber SN, Knutson B, 2010. The reward circuit: linking primate anatomy and human imaging. Neuropsychopharmacology 35 (1). doi:10.1038/npp.2009.129. PMC3055449 19812543

[R22] Hale AT, Stonko DP, Wang L, Strother MK, Chambless LB, 2018. Machine learning analyses can differentiate meningioma grade by features on magnetic resonance imaging. Neurosurg. Focus 45 (5). doi:10.3171/2018.8.FOCUS18191. 30453458

[R23] Hasan AM, Jalab HA, Meziane F, Kahtan H, Al-Ahmad AS, 2019. Combining deep and handcrafted image features for MRI brain scan classification. IEEE Access 7. doi:10.1109/ACCESS.2019.2922691.

[R24] Hooks MS, Kalivas PW, 1994. Involvement of dopamine and excitatory amino acid transmission in novelty- induced motor activity. J. Pharmacol. Exp. Ther 269 (3). 7516970

[R25] Hughes RN, Bakhurin KI, Petter EA, Watson GDR, Kim N, Friedman AD, Yin HH, 2020. Ventral tegmental dopamine neurons control the impulse vector during motivated behavior. Curr. Biol 30 (14). doi:10.1016/j.cub.2020.05.003. PMC7590264 32470362

[R26] Ikemoto S, 2007. Dopamine reward circuitry: two projection systems from the ventral midbrain to the nucleus accumbens-olfactory tubercle complex. Brain Res. Rev 56 (1). doi:10.1016/j.brainresrev.2007.05.004. PMC2134972 17574681

[R27] Jacobs H, Becker J, Kwong K, Johnson K, 2022. High-resolution structural brainstem nuclei: locus coeruleus integrity as potential biomarker of Alzheimer’s disease-related phenotypes. Bio. Psychiatry 91 (9), S31.

[R28] Kim HGR, Malik AN, Mikhael JG, Bech P, Tsutsui-Kimura I, Sun F, Zhang Y, Li Y, Watabe-Uchida M, Gershman SJ, Uchida N, 2020. A unified framework for dopamine signals across timescales. Cell 183 (6). doi:10.1016/j.cell.2020.11.013. PMC7736562 33248024

[R29] Klein-Flügge MC, Kennerley SW, Friston K, Bestmann S, 2016. Neural signatures of value comparison in human cingulate cortex during decisions requiring an effort-reward trade-off. J. Neurosci 36 (39). doi:10.1523/JNEUROSCI.0292-16.2016. PMC5039251 27683898

[R30] Klein-Flügge MC, Kennerley SW, Saraiva AC, Penny WD, Bestmann S, 2015. Behavioral modeling of human choices reveals dissociable effects of physical effort and temporal delay on reward devaluation. PLoS Comput. Biol 11 (3). doi:10.1371/journal.pcbi.1004116. PMC4376637 25816114

[R31] Knörle R, 2018. Neuromelanin in Parkinson’s disease: from Fenton reaction to calcium signaling. Neurotox. Res 33 (2). doi:10.1007/s12640-017-9804-z. 28879408

[R32] Kokane SS, Perrotti LI, 2020. Sex Differences and the role of estradiol in mesolimbic reward circuits and vulnerability to cocaine and opiate addiction. Front. Behav. Neurosci 14. doi:10.3389/fnbeh.2020.00074. PMC7251038 32508605

[R33] Kritzer MF, Creutz LM, 2008. Region and sex differences in constituent dopamine neurons and immunoreactivity for intracellular estrogen and androgen receptors in mesocortical projections in rats. J. Neurosci 28 (38). doi:10.1523/JNEUROSCI.2637-08.2008. PMC2613180 18799684

[R34] Kutlu MG, Zachry JE, Melugin PR, Cajigas SA, Chevee MF, Kelly SJ, Kutlu B, Tian L, Siciliano CA, Calipari ES, 2021. Dopamine release in the nucleus accumbens core signals perceived saliency. Curr. Biol 31 (21). doi:10.1016/j.cub.2021.08.052. PMC9084920 34529938

[R35] Lamers F, van Oppen P, Comijs HC, Smit JH, Spinhoven P, van Balkom AJLM, Nolen WA, Zitman FG, Beekman ATF, Penninx BWJH, 2011. Comorbidity patterns of anxiety and depressive disorders in a large cohort study: the Netherlands Study of Depression and Anxiety (NESDA). J. Clin. Psychiatry 72 (3). doi:10.4088/JCP.10m06176blu. 21294994

[R36] Lammel S, Lim BK, Malenka RC, 2014. Reward and aversion in a heterogeneous midbrain dopamine system. Neuropharmacology 76 (PART B). doi:10.1016/j.neuropharm.2013.03.019. PMC3778102 23578393

[R37] Langley J, Huddleston DE, Chen X, Sedlacik J, Zachariah N, Hu X, 2015. A multicontrast approach for comprehensive imaging of substantia nigra. Neuroimage 112. doi:10.1016/j.neuroimage.2015.02.045. PMC4415274 25731994

[R38] Lehéricy S, Bardinet E, Poupon C, Vidailhet M, François C, 2014. 7 tesla magnetic resonance imaging: a closer look at substantia nigra anatomy in Parkinson’s disease. Mov. Disord 29 (13). doi:10.1002/mds.26043. 25308960

[R39] Marques JP, Kober T, Krueger G, van der Zwaag W, van de Moortele PF, Gruetter R, 2010. MP2RAGE, a self bias-field corrected sequence for improved segmentation and T1-mapping at high field. Neuroimage 49 (2). doi:10.1016/j.neuroimage.2009.10.002. 19819338

[R40] McArthur S, McHale E, Gillies GE, 2007. The size and distribution of midbrain dopaminergic populations are permanently altered by perinatal glucocorticoid exposure in a sex- region- and time-specific manner. Neuropsychopharmacology 32 (7). doi:10.1038/sj.npp.1301277. 17164817

[R41] Mehrotra R, Ansari MA, Agrawal R, Anand RS, 2020. A transfer learning approach for AI-based classification of brain tumors. Mach. Learn. Appl 2. doi:10.1016/j.mlwa.2020.100003.

[R42] Mohebi A, Pettibone JR, Hamid AA, Wong JMT, Vinson LT, Patriarchi T, Tian L, Kennedy RT, Berke JD, 2019. Dissociable dopamine dynamics for learning and motivation. Nature 570 (7759). doi:10.1038/s41586-019-1235-y. PMC6555489 31118513

[R43] Morris LS, Kundu P, Costi S, Collins A, Schneider M, Verma G, Balchandani P, Murrough JW, 2019. Ultra-high field MRI reveals mood-related circuit disturbances in depression: a comparison between 3-Tesla and 7-Tesla. Transl. Psychiatry 9 (1). doi:10.1038/s41398-019-0425-6. PMC6377652 30770788

[R44] Morris LS, Norbury A, Smith DA, Harrison NA, Voon V, Murrough JW, 2020. Dissociating self-generated volition from externally-generated motivation. PLoS One 15 (5). doi:10.1371/journal.pone.0232949. PMC7236980 32428020

[R45] Morris LS, Tan A, Smith DA, Grehl M, Han-Huang K, Naidich TP, Charney DS, Balchandani P, Murrough JW, Kundu P, 2020. Sub-millimeter variation in human locus coeruleus is associated with dimensional measures of psychopathology: an *in vivo* ultra-high field 7-Tesla MRI study. NeuroImage Clin. 25. doi:10.1016/j.nicl.2019.102148. PMC7037543 32097890

[R46] Morris LS, Grehl MM, Rutter SB, Mehta M, Westwater ML, 2022. On what motivates us: a detailed review of intrinsic v. extrinsic motivation. Psychol. Med 1–16. 10.1017/S0033291722001611 PMC9340849 35796023

[R47] Müller T, Klein-Flügge MC, Manohar SG, Husain M, Apps MAJ, 2021. Neural and computational mechanisms of momentary fatigue and persistence in effort-based choice. Nat. Commun 12 (1). doi:10.1038/s41467-021-24927-7. PMC8319292 34321478

[R48] Murayama K, Matsumoto M, Izuma K, Matsumoto K, 2010. Neural basis of the undermining effect of monetary reward on intrinsic motivation. Proc. Natl. Acad. Sci. USA 107 (49). doi:10.1073/pnas.1013305107. PMC3000299 21078974

[R49] Murayama K, Matsumoto M, Izuma K, Sugiura A, Ryan RM, Deci EL, Matsumoto K, 2015. How self-determined choice facilitates performance: a key role of the ventromedial prefrontal cortex. Cereb. Cortex 25 (5). doi:10.1093/cercor/bht317. 24297329

[R50] Nair-Roberts RG, Chatelain-Badie SD, Benson E, White-Cooper H, Bolam JP, Ungless MA, 2008. Stereological estimates of dopaminergic, GABAergic and glutamatergic neurons in the ventral tegmental area, substantia nigra and retrorubral field in the rat. Neuroscience 152 (4). doi:10.1016/j.neuroscience.2008.01.046. PMC2575227 18355970

[R51] Nobin A, Bjorklund A, 1973. Topography of the monoamine neuron systems in the human brain as revealed in fetuses. Acta Physiol. Scand 388 Sup. 4521068

[R52] Panksepp J, & Wilson CG (2016). Brain SEEKING circuitry in neuroeconomics: a unifying hypothesis for the role of dopamine-energized arousal of the medial forebrain bundle in enthusiasm-guiding decision-making. Part of the Studies in Neuroscience, Psychology and Behavioral Economics book series (SNPBE). doi:10.1007/978-3-642-35923-1_13.

[R53] Pauli WM, Nili AN, Michael Tyszka J, 2018. Data descriptor: a high-resolution probabilistic in vivo atlas of human subcortical brain nuclei. Sci. Data 5. doi:10.1038/sdata.2018.63. PMC5903366 29664465

[R54] Pedregosa F, Varoquaux G, Gramfort A, Michel V, Thirion B, Grisel O, Blondel M, Prettenhofer P, Weiss R, Dubourg V, Vanderplas J, Passos A, Cournapeau D, Brucher M, Perrot M, Duchesnay É, 2011. Scikit-learn: machine learning in Python. J. Mach. Learn. Res 12.

[R55] Priovoulos N, Jacobs HIL, Ivanov D, Uludağ K, Verhey FRJ, Poser BA, 2018. High-resolution in vivo imaging of human locus coeruleus by magnetization transfer MRI at 3T and 7T. Neuroimage 168. doi:10.1016/j.neuroimage.2017.07.045. 28743460

[R56] Priovoulos N, van Boxel SCJ, Jacobs HIL, Poser BA, Uludag K, Verhey FRJ, Ivanov D, 2020. Unraveling the contributions to the neuromelanin-MRI contrast. Brain Struct. Funct 225 (9). doi:10.1007/s00429-020-02153-z. PMC7674382 33090274

[R57] Qi G, Zhang P, Li T, Li M, Zhang Q, He F, Zhang L, Cai H, Lv X, Qiao H, Chen X, Ming J, Tian B, 2022. NAc-VTA circuit underlies emotional stress-induced anxiety-like behavior in the three-chamber vicarious social defeat stress mouse model. Nat Commun. 13 (1), 577. doi:10.1038/s41467-022-28190-2. 35102141 PMC8804001

[R58] Reyes S, Fu Y, Double K, Thompson L, Kirik D, Paxinos G, Halliday GM, 2012. GIRK2 expression in dopamine neurons of the substantia nigra and ventral tegmental area. J. Comp. Neurol 520 (12). doi:10.1002/cne.23051. 22252428

[R59] Rincón-Cortés M, Grace AA, 2017. Sex-dependent effects of stress on immobility behavior and VTA dopamine neuron activity: modulation by ketamine. Int. J. Neuropsychopharmacol 20 (10). doi:10.1093/ijnp/pyx048. PMC5632304 28591782

[R60] Rusheen AE, Gee TA, Jang DP, Blaha CD, Bennet KE, Lee KH, Heien ML, Oh Y, 2020. Evaluation of electrochemical methods for tonic dopamine detection *in vivo*. TrAC Trends Anal. Chem 132. doi:10.1016/j.trac.2020.116049. PMC7885180 33597790

[R61] Salamone JD, Pardo M, Yohn SE, López-Cruz L, Sanmiguel N, Correa M, 2016. Mesolimbic dopamine and the regulation of motivated behavior. Curr. Top. Behav. Neurosci 27. doi:10.1007/7854_2015_383. 26323245

[R62] Schlaepfer TE, Bewernick B, Kayser S, Lenz D, 2011. Modulating affect, cognition, and behavior - prospects of deep brain stimulation for treatment-resistant psychiatric disorders. Front. Integr. Neurosci 5. doi:10.3389/fnint.2011.00029. PMC3125515 21738500

[R63] Schultz W, 1998. Predictive reward signal of dopamine neurons. J. Neurophysiol 80 (1). doi:10.1152/jn.1998.80.1.1. 9658025

[R64] Starkweather CK, Uchida N, 2021. Dopamine signals as temporal difference errors: recent advances. Curr. Opin. Neurobiol 67. doi:10.1016/j.conb.2020.08.014. PMC8107188 33186815

[R65] Steiger TK, Bunzeck N, 2017. Reward dependent invigoration relates to theta oscillations and is predicted by dopaminergic midbrain integrity in healthy elderly. Front. Aging Neurosci 9 (JAN). doi:10.3389/fnagi.2017.00001. PMC5258705 28174533

[R66] Treadway MT, Buckholtz JW, Schwartzman AN, Lambert WE, Zald DH, 2009. Worth the “EEfRT”? The effort expenditure for rewards task as an objective measure of motivation and anhedonia. PLoS One 4 (8). doi:10.1371/journal.pone.0006598. PMC2720457 19672310

[R67] Trutti AC, Mulder MJ, Hommel B, Forstmann BU, 2019. Functional neuroanatomical review of the ventral tegmental area. Neuroimage 191. doi:10.1016/j.neuroimage.2019.01.062. 30710678

[R68] Tzschentke TM, 2000. The medial prefrontal cortex as a part of the brain reward system. Amino Acids 19 (1). doi:10.1007/s007260070051. 11026491

[R69] van Domburg PH, ten Donkelaar HJ, 1991. The human substantia nigra and ventral tegmental area. A neuroanatomical study with notes on aging and aging diseases. Adv. Anat. Embryol. Cell Biol 121. doi:10.1016/0022-510x(91)90316-y. 2053466

[R70] Walsh JJ, Han MH, 2014. The heterogeneity of ventral tegmental area neurons: projection functions in a mood-related context. Neuroscience 282. doi:10.1016/j.neuroscience.2014.06.006. PMC4339667 24931766

[R71] Watanabe Y, Tanaka H, Tsukabe A, Kunitomi Y, Nishizawa M, Hashimoto R, Yamamori H, Fujimoto M, Fukunaga M, Tomiyama N, 2014. Neuromelanin magnetic resonance imaging reveals increased dopaminergic neuron activity in the substantia nigra of patients with schizophrenia. PLoS One 9 (8). doi:10.1371/journal.pone.0104619. PMC4128756 25111500

[R72] Yamashita F, Sasaki M, Fukumoto K, Otsuka K, Uwano I, Kameda H, Endoh J, Sakai A, 2016. Detection of changes in the ventral tegmental area of patients with schizophrenia using neuromelanin-sensitive MRI. Neuroreport 27 (5). doi:10.1097/WNR.0000000000000530. 26901057

[R73] Yu C, Li X, Wang S, Zhang W, 2016. Teacher autonomy support reduces adolescent anxiety and depression: an 18-month longitudinal study. J. Adolesc 49. doi:10.1016/j.adolescence.2016.03.001. 27042976

[R74] Zecca L, Zucca FA, Wilms H, Sulzer D, 2003. Neuromelanin of the substantia nigra: a neuronal black hole with protective and toxic characteristics. Trends Neurosci. 26 (11). doi:10.1016/j.tins.2003.08.009. 14585596

[R75] Zucca FA, Basso E, Cupaioli FA, Ferrari E, Sulzer D, Casella L, Zecca L, 2014. Neuromelanin of the human substantia nigra: an update. Neurotox. Res 25 (1). doi:10.1007/s12640-013-9435-y. 24155156

