# Supplementary figures and images for: Ventral tegmental area integrity measured with high-resolution 7-Tesla MRI relates to motivation across depression and anxiety diagnoses
Source: Neuroimage. Author manuscript; Available in PMC 2022 Dec 30. (PMC9801251; doi:10.1016/j.neuroimage.2022.119704)

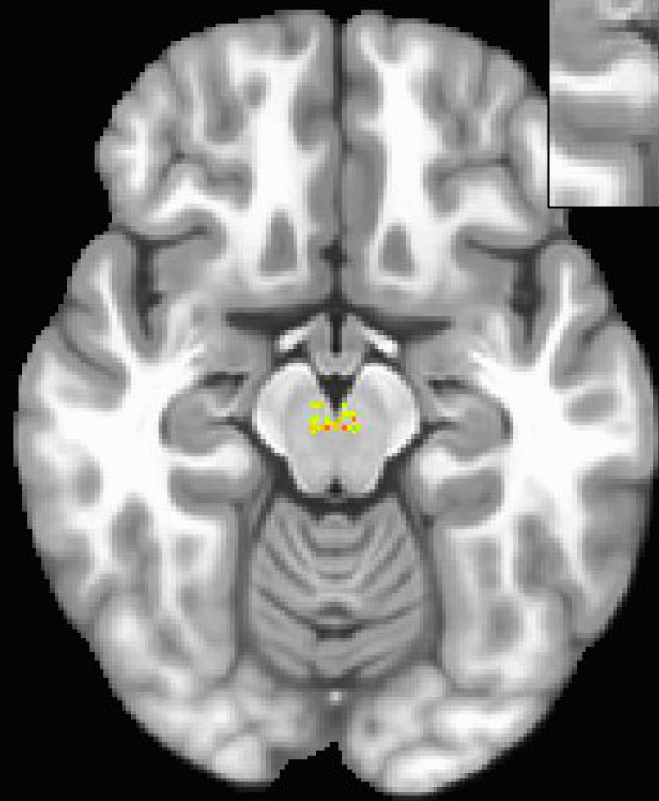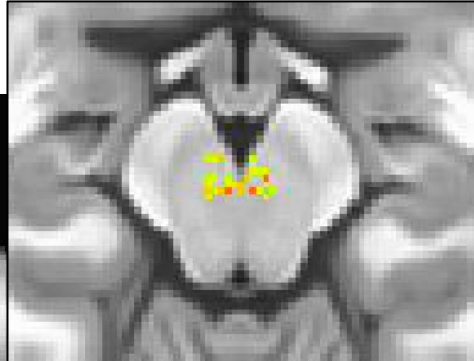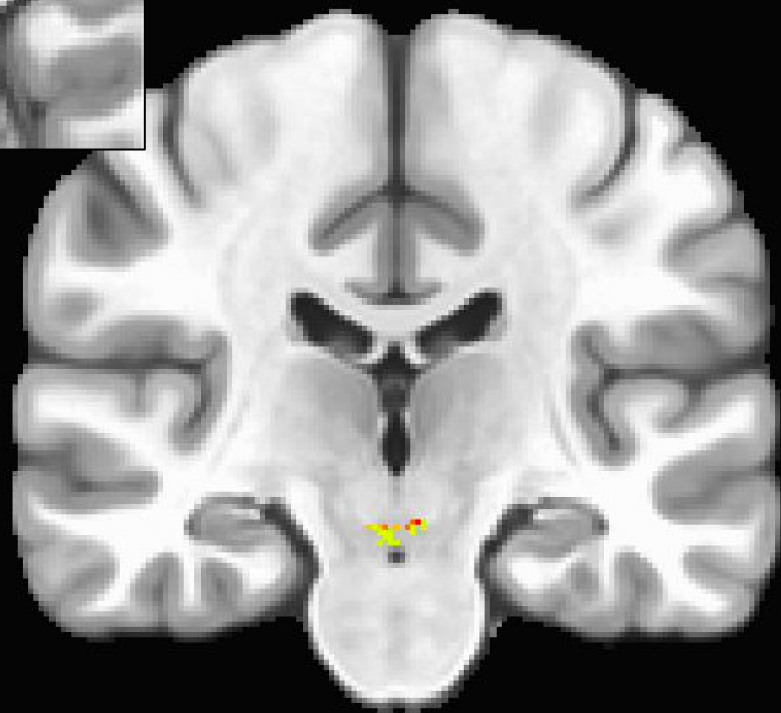

Supplement: 1 [file NIHMS1860167-supplement-1.pdf]

A.

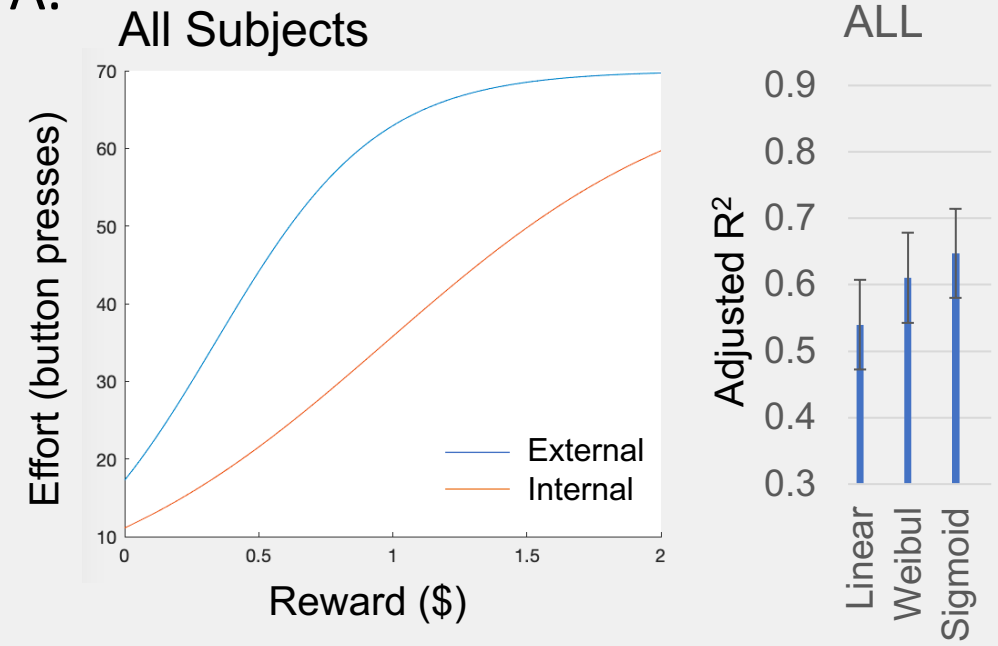

B.

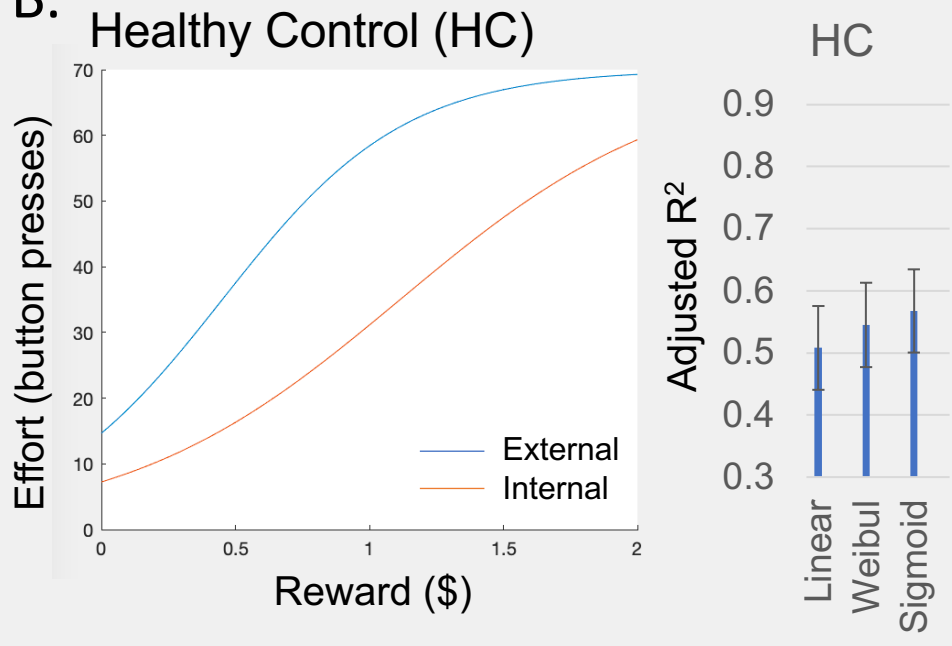

C.

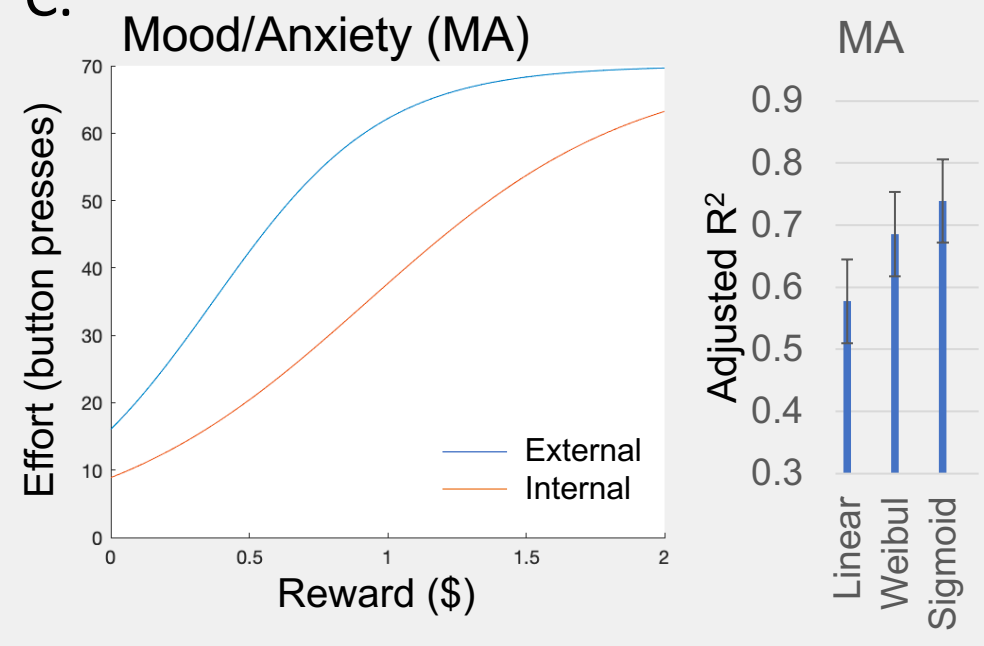

D.

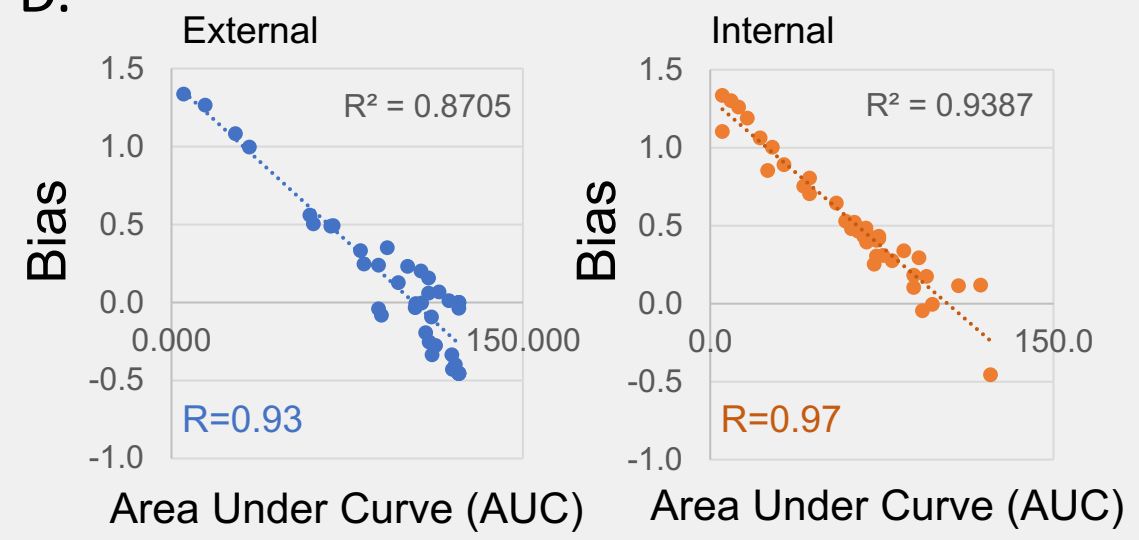

Supplement: 2 [file NIHMS1860167-supplement-2.pdf]
