# Supplementary material for: Ventral tegmental area integrity measured with high-resolution 7-Tesla MRI relates to motivation across depression and anxiety diagnoses
Source: Neuroimage. Author manuscript; Available in PMC 2022 Dec 30. (PMC9801251; doi:10.1016/j.neuroimage.2022.119704)
Supplement: 3 [file NIHMS1860167-supplement-3.docx]

**Supplementary Materials**

**Supplementary Methods**

To determine the relationship between effort and reward in this study, a sigmoid model was fit to the choice data, given a range of previous studies both from our laboratory (Morris et al., 2020) and others (Klein-Flugge, Kennerley et al. 2015, Chong, Bonnelle et al. 2016) using similar tasks and showing that the sigmoid model outperforms other models. Other commonly used models, including linear and Weibull models were also tested and compared to the sigmoid. Linear, sigmoid and Weibull functions were fit to the effort-by-reward discount curves for each condition for subject using the variational Bayes approach to model inversion implemented in the VBA toolbox (available at mbb-team.github.io/VBA-toolbox), run in MATLAB R2019a. Similar to our previous findings, the sigmoid model outperformed these other models in terms of percentage of variance explained and corrected model accuracy, accounting for number of model parameters or number of predictors in the model (adjusted-R^2^, see Supplementary Figure 2). This is in line with other published studies to quantify individual differences in response against parametric modulation of an experimental factor (e.g., reward, perceptual difference etc.). In particular, (Klein-Flugge, Kennerley et al. 2015) directly test different models and converge also on the sigmoid function as the best fitting for effort discounting data, similar to the current work. Altogether, both from the literature, our previous findings, and this current finding, we confirm that sigmoid curve is a relatively simple two-parameter curve with good exceedance probability in general for effort x reward discounting tasks and is generally an acceptable curve-fit.

Sigmoid:

y = c * 1./(1 + exp(-x – bias)*sigma)))

where the bias parameter governs the left-right translation of the function (bias against exerting effort, for a given amount of reward), sigma is the gradient governing reward sensitivity (increase in effort per increase in unit reward offered).

**Supplementary Figures**

**Supplementary Figure 1.** **Machine-learning based data-driven segmentations of ventral tegmental area in subjects with mood and anxiety disorders.** Composite group image of VTA segmentations produced by a mixture of supervised and unsupervised models based on high-resolution 7T 400 µm^3^ magnetization transfer contrast MRI for subjects with mood and anxiety disorders.

**Supplementary Figure 2. Sigmoid model fit curves for each group for external and internal task conditions.** Effort by reward discount curves as fit by a sigmoid model are presented where the blue line is the curve generated using the mean posterior parameter values for that participant (sigmoid model) for the external (“extrinsic”) condition, and the orange line indicates the internal (“intrinsic”) condition. Adjusted-R^2^ model fit values are plotted for the sigmoid model as well as other popular models such as linear and Weibull. Plots are separated by all subjects combined (A), the healthy control group (B) and the mood and anxiety disorders group (C). The main parameter of interest obtained from the sigmoid model that represents motivational tone – bias – is also plotted against the area under the curve (AUC) for all subjects for each condition (D), demonstrating a tight relationship between these two measures.

**Supplementary Table**

|  | VTA Signal Intensity | VTA Volume | Anhedonia (ant.) | Anhedonia (con.) | Anxiety | External Bias | Internal Bias | External Reward Sensitivity | Internal Reward Sensitivity |
| --- | --- | --- | --- | --- | --- | --- | --- | --- | --- |
| VTA Signal Intensity |  |  |  |  |  |  |  |  |  |
| VTA Volume | >0.05 |  |  |  |  |  |  |  |  |
| Anhedonia (ant.) | >0.05 | >0.05 |  |  |  |  |  |  |  |
| Anhedonia (con.) | >0.05 | >0.05 | p<0.001 |  |  |  |  |  |  |
| Anxiety | >0.05 | >0.05 | >0.05 | >0.05 |  |  |  |  |  |
| External Bias | p=0.041 | >0.05 | *p=0.016* | >0.05 | p<0.001 |  |  |  |  |
| Internal Bias | >0.05 | >0.05 | >0.05 | >0.05 | >0.05 | p<0.001 |  |  |  |
| External Reward Sensitivity | >0.05 | *p=0.026* | >0.05 | >0.05 | *p<0.001* | >0.05 | >0.05 |  |  |
| Internal Reward Sensitivity | >0.05 | >0.05 | p=0.049 | >0.05 | *p<0.001* | >0.05 | >0.05 | p<0.001 |  |

**Supplementary Table 1. All correlations between cognitive task, self-report and ventral tegmental area (VTA) measures for all subjects.** Anhedonia (ant., anticipatory, and con., consummatory) was measured via the Temporal Experience of Please Scale (TEPS), anxiety via the State-Trait Cognitive and Somatic Anxiety (STICSA) total score. External and internal sigma and bias parameters were obtained from the cognitive effort discounting task, the Internal-External Motivation Task (IMT). Ventral tegmental area (VTA) normalized measures of volume and signal intensity were obtained from a 7-Tesla magnetization transfer scan with computational modeling. P values are displayed for each uncorrected pairwise correlation, with negative correlations depicted in italics.
